# Black Cohosh and Liver Toxicity: Is There a Relationship?

**DOI:** 10.1155/2014/860614

**Published:** 2014-06-30

**Authors:** Mohammed Muqeet Adnan, Muhammad Khan, Syed Hashmi, Muhammad Hamza, Sufyan AbdulMujeeb, Syed Amer

**Affiliations:** ^1^Department of Internal Medicine, University of Oklahoma Health Sciences Center, Oklahoma City, OK 73112, USA; ^2^Dow Medical College, Karachi 74200, Pakistan; ^3^University of Illinois at Chicago, Chicago, IL 60680, USA; ^4^Mayo Clinic Arizona, Phoenix, AZ 85054, USA

## Abstract

Herbal supplements are commonly used by patients for various problems. It is a well-known fact that most patients do not tell their physicians about the use of herbal supplements unless they are specifically asked. As a result, sometimes important information regarding drug side effects is missed in history taking. During our literature search, we found several retrospective studies and other meta-analyses that claim a lacking or weak link between black cohosh use and hepatotoxicity. We present a case of a 44-year-old female who developed subacute liver injury demonstrated on a CT scan and liver biopsy within a month of using the drug to resolve her hot flashes and discuss a possible temporal and causal association between black cohosh use and liver disease. Since the patient was not taking any other drugs, we concluded that the acute liver injury was caused by the use of black cohosh. We agree with the United States Pharmacopeia recommendations that a cautionary warning about hepatotoxicity should be labeled on the drug package.

## 1. Case

A 44-year-old female with no significant medical history presented with complaints of having painless jaundice for one month. She went to her primary care physician's office where initial workup revealed that she had elevated liver function tests (LFTs). She was noted to have normal LFTs on her prior lab works. Workup for viral and autoimmune hepatitis was negative. She was given a trial of steroids on an outpatient basis without much improvement; later, she was admitted for worsening jaundice. She denied history of alcohol intake, IV drug use, unprotected sex, recent travel outside the United States, NSAID ingestion, and blood transfusions. The review of systems was positive for generalized itching, arthralgia, and fatigue. She did not complain of abdominal pain, fever, chills, nausea, vomiting, or diarrhea. During the inpatient workup, she reported that she started taking black cohosh about a month ago to alleviate her menopausal symptoms. Her examination was remarkable for marked scleral icterus and jaundiced skin. Upon admission, her LFTs showed total bilirubin: 20 mg/dL, aspartate transaminase: 420 U, alanine transaminase: 215 U/L, alkaline phosphatase: 201 U/L, platelets: 135 k/mm^3^, international normalized ratio: 1.2, and albumin: 2.4 g/dL. An ultrasound of the abdomen showed nodular contour of the liver consistent with cirrhosis. Further workup ruled out Wilson's disease, hemochromatosis, primary biliary cirrhosis, and autoimmune hepatitis. Hepatitis A, B, C antigens and antibodies were negative ruling out acute or chronic viral hepatitis. The EBV, CMV, and HSV IgM antibodies and PCR test were negative.

**Figure 1:**
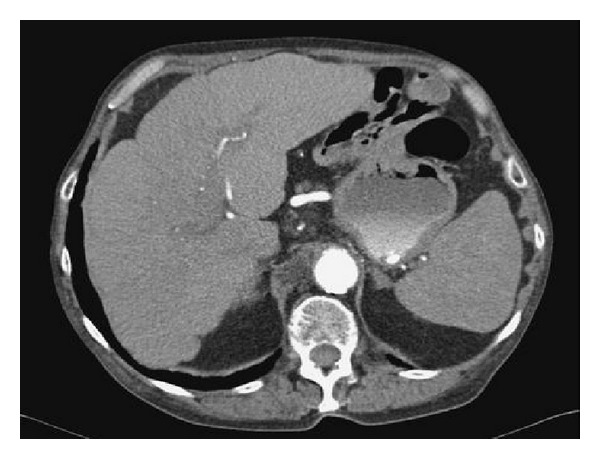
CT scan showing nodular liver contour, indicative of early hepatic injury and cirrhosis.

**Figure 2:**
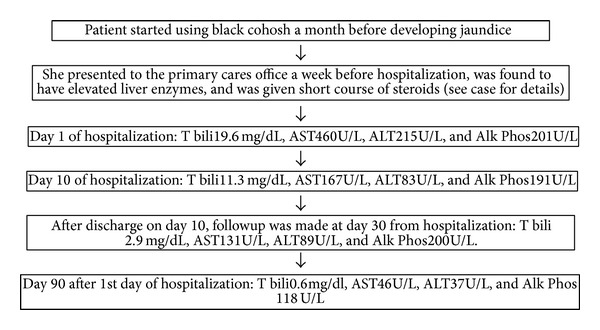
Flow chart showing sequence of events. The black cohosh dechallenge was begun from day 1 of hospitalization. LFTs normalized within 90 days.

A liver biopsy was performed, which showed a histologic pattern consistent with cholestasis and hepatocellular injury (see Figure 1). Given the patient's history of black cohosh use and the timing of her abnormal liver chemistries, it was clinically evident that the culprit agent was black cohosh. Her symptoms improved and her LFTs normalized after she stopped taking black cohosh. (see Figure 2).

Based on the CIOMS criteria our patient scored a +9 and hence we think that the liver injury was highly probable from the use of black cohosh [1, 2].

## 2. Discussion

Black cohosh (*Actaea racemosa* a. k. a.* Cimicifuga racemosa*) is a herbal rhizome generally used to treat hot flashes and menopausal symptoms in women instead of hormone replacement therapy [3]. Hepatotoxicity from black cohosh has been documented in many case reports, including some that needed transplantation [4–9]. Black cohosh contains catechols, which when activated can convert to electrophilic quinones and cause free radical mediated injury [3]. Teschke et al. have reported about 69 cases of hepatotoxicity from black cohosh in their paper and say that the evidence of a causal relationship is weak, even nonexistent [10]. Multiple retrospective studies, prospective studies, and meta-analyses done in the past by Teschke et al., Firenzouli et al., and Naser et al. claim that they found a lack of evidence demonstrating causality between black cohosh and liver injury [3, 10–12]. In their retrospective studies, they all claim either weak or insufficient evidence of black cohosh use causing hepatotoxicity.

We present a 44-year-old female with a short course of black cohosh intake that presented with painless jaundice. The CT scan clearly shows a nodular appearance consistent with liver cirrhosis. Multiple causes of liver cirrhosis were ruled out, as described in the case above. There were no other medications that the patient was taking at the same time. Remarkable improvement in the liver function after stopping the drug clearly demonstrates that there is a causal relationship. Our evidence may not be as strong as that which is presented by Teschke et al., Firenzouli et al., or Naser et al. [3, 10, 12], but we have supporting case reports, such as those found in the paper published by Lim et al., in which black cohosh is described as causing fulminant hepatic failure to the extent that the patient needed transplantation [4]. The essence of this paper is to not discourage the use of black cohosh but to serve as a warning for patients to use it with caution, especially those who are prone to develop liver diseases. An increased likelihood of developing liver disease can only be detected by measuring serial liver function tests while using the drug. Based on the hepatotoxicity case reports from black cohosh, we agree with Teschke et al. that the evidence is weak to support causality, but the possible causal relationship cannot be completely ignored [13]. Mahady et al. looked at 30 case reports and did not find sufficient evidence of a causal relationship between hepatotoxicity and black cohosh use; however, they suggest that a cautionary warning should be placed on the label [14].

Firenzouli et al. claim that herbal medicine alone cannot be the cause for the side effects; multiple substances, such as heavy metals and other toxins, usually coexist in combination with herbal medicine to cause side effects [3]. Though we are unable to definitively report whether the patient was taking pure* A. racemosa* extract or a combination of herbal products that contained other substances, based upon the medical history provided by the patient and the subacute presentation, we conclude that there is a possible causal relationship between hepatotoxicity and black cohosh use. Despite the fact that multiple studies in the past claim that there was no correlation between black cohosh use and hepatotoxicity, we think the subacute drug induced hepatitis in our patient who had recently started taking this herbal medicine was the cause of her acute hepatic injury.

## 3. Limitations

We do not know the exact dose and the purity of the herbal product that the patient was taking for her menopausal symptoms, and hence blaming the liver injury on* A. racemosa* extract alone would be very difficult. Health Canada published an update about liver toxicity from black cohosh and 5 out of the 6 cases that they report did not contain authentic black cohosh [15]. Unfortunately, we were not able to analyze the patient's herbal black cohosh extract.

## 4. Conclusion

Causality attribution for a herb in cases of assumed herb induced liver injury requires clear identification of the incriminated herbal product used by the patient and information of the daily dose. Stricter FDA and government regulations need to be applied to the purity of herbal remedies like black cohosh, as the impurities in them could be responsible for their adverse effects.
